# Grand Challenges, Covid‐19 and the Future of Organizational Scholarship

**DOI:** 10.1111/joms.12647

**Published:** 2020-10-19

**Authors:** Jennifer Howard‐Grenville

**Affiliations:** ^1^ University of Cambridge

**Keywords:** grand challenges, organization studies, organization theory, organizational scholarship, sustainability

Grand challenges are ‘formulations of global problems that can be plausibly addressed through coordinated and collaborative effort’ (George et al., 2016, p. 1880). By this definition, the Covid‐19 pandemic certainly registers as a grand challenge. It is a type of problem scientists have been warning about for decades – a pandemic unleashed when a virus jumps from animals to humans, in part due to habitat loss (IPBES, 2019). The pandemic’s global reach has no bounds; its economic, social, and health consequences affect us all. We all hope this problem can be plausibly addressed through coordinated and collaborative effort. Indeed, the myriad researchers working globally to develop a vaccine suggest extensive coordinated – though not exclusively collaborative (Spinney, 2020) – effort.

Could there be a more pressing and urgent grand challenge than this? And, what might its lessons be for how organisational scholars engage in work that seeks to understand and tackle societal grand challenges?

In this essay, I reflect on the grand challenges discourse and how it has and should be taken up in our field. I revisit our 2016 *AMJ* essay, (George et al., 2016), where we articulated the importance of societal grand challenges for management research and offered a framework for how scholars might conduct such research. The Covid‐19 pandemic offers an opportunity to reconsider our original messages.

## The Nature and Origin of Grand Challenges

Organisational scholars were relatively late in talking explicitly about grand challenges (Colquitt and George, 2011), but attention increased considerably in the last 5 years (Ferraro et al., 2015; George et al., 2016). Grand challenges are said to originate with the articulation in 1900 by a German mathematician, David Hilbert, of 23 mathematical problems that, once solved, would enable further progress in the field. This origin story gained momentum when Bill Gates referred to it in announcing his 2003 Grand Challenges in Global Health Initiative, which set out 14 specific scientific goals.

The origins of the concept of grand challenges embed certain assumptions that persist. First, grand challenges were framed as discrete and *tractable* problems. In mathematics, then, in other sciences, groups designated specific problems, often with time frames and money attached, whose solution would unlock further progress (Omenn, 2006). Second, grand challenges were framed explicitly to *attract attention and focus resources* on salient problems. Challenges motivated researchers individually and collectively by articulating a purpose for their work. They also communicated with ‘journalists, the public, and their elected representatives’ to ‘show the added value of further major investments in research’ (Omenn, 2006, p. 1696).

While articulating them is partly strategic, the discourse around grand challenges captures and reflects – and might even perform – a shift in how we think about the role of research in society in the 21^st^ century, and it is a label that has stuck.

## Grand Challenges and Organizational Scholarship

So, what does this mean for our scholarship, and how has the pandemic shed light on how we undertake this work?

### Why We Cannot Have a List – and Should not Aspire to

Calls for addressing grand challenges in organizational and management research have strongly resonated. Research under the label has proliferated – from topics we would expect like climate change, pollution, and inequality (e.g., Mair et al., 2016; Porter et al., 2020; Wright and Nyberg, 2017), to less expected ones like sport and population health (Inoue et al., 2019). With this explosion of activity, organizational scholars have used the grand challenges label in different ways, which some argue risks ‘conflat[ing] qualitatively distinct types of phenomena, levels of analysis, and scales/scopes of issues’, potentially lessening the work’s impact (Brammer et al., 2019, p. 518).

Is the answer a tighter definition, or even the articulation of our own list of grand challenges for management research? The nature of the grand challenges now at the forefront of attention in our field and others are increasingly belying the original characteristics of discreteness and tractability. There is a stark difference between 23 mathematical problems or even the scientific challenge of developing vaccines that do not require refrigeration (a 2003 Gates Foundation grand challenge) and issues like mitigating climate change, or preventing pandemics. Even taking a tighter focus on a specific challenge – like developing a safe and effective vaccine for the coronavirus pandemic – illustrates the risk of focusing on one seemingly tractable aspect of an extraordinarily complex phenomenon. While developing a vaccine is certainly a critical part of the technical quiver that may help end the pandemic, questions we never knew to ask are also arising as more is observed about how the virus behaves in varied settings. In South Africa, for example, death rates are seven times lower than those in the UK and infections (even if under‐reported) are bafflingly low, leading experts to speculate that the very conditions thought to fuel a massive outbreak – poverty and crowded living – may be protective, having enabled widespread exposure to earlier coronaviruses that confer some immunity (Harding, 2020).

Given their defiance of straightforward solutions or even stable specifications, we need to recognize grand challenges as ‘seemingly intractable’ puzzles (Ferraro, et al., 2015, p. 367). Ferraro and co‐authors characterize grand challenges as inherently i) complex – interactions and constraints are not only multiple but only partially visible to any actor at any time – ii) uncertain – decisions have to be made in spite of unknowable future consequences or preferences – and iii) evaluative – there is no single set of criteria upon which people agree about the problem let alone solution.^[^ ^[1]^ ^]^ Even the UN acknowledges the ‘trade‐offs and tough choices … at the heart of’ working towards the 17 Sustainable Development Goals (UN, 2019, p. 3), regarded as the most encompassing societal grand challenges.

Organizational scholars, then, should move away from assumptions of tractability – and certainly any need for a list – and be transparent about the characteristics of complexity, uncertainty, and evaluativity in our work. Giving explicit consideration to the scope and scale of issues, variety of actors involved, and degree of complexity (Brammer et al., 2019) will help establish boundary conditions of our findings. Most importantly, we need to embrace a mode of inquiry into grand challenges that is more exploratory, pluralistic, and driven by debate than one imprinted from its origins in mathematics and science.

### What We Should Do

Without a list of tractable challenges, where are we? We are likely squarely in our comfort zone as organizational scholars, for we are rarely content experts on any grand challenges per se (Claus et al., 2019) but rather experts on understanding multilevel processes of organizing and their interactions and consequences, forms of advocacy, engagement, or obfuscation that shape issue salience and organizational action or inaction, and ways in which complex interorganizational relationships play out with far‐reaching consequences. We are a field cobbled together from various disciplinary roots, so our theories tackle processes at diverse levels of analysis, through different core assumptions, and seek to explain diverse questions. We can embrace these characteristics as strengths for understanding the complex processes that underpin grand challenges’ persistence and mitigation.

First, a multilevel perspective on phenomena enables us to trace and appreciate interactions across levels, which are critical to how grand challenges arise and how they might be tackled (Howard‐Grenville et al., 2019, p. 357). Second, the mechanisms we use to explain these phenomena increasingly capture their inherent complexity. For example, paradox, systems, and robust action (e.g., Porter et al., 2020; Whiteman and Yumashev, 2018), inform how people make sense of, frame, and act on grand challenges. Understanding how, when and why issues become articulated as grand challenges, whose interests this serves, and how their salience ebbs and flows is critically important to working towards mitigating them. Put differently, unlike other scholarly disciplines, we might consider ourselves process – versus content – experts on grand challenges. What more can we add?

In the 2016 essay, we presented a framework for thinking about grand challenges (George et al., 2016). We noted that, while others typically articulate challenges, we can study how needs and aspirations interact with barriers and opportunities at the individual, organizational and institutional level to shape action. While it was never intended as a process model, this framework nonetheless visually depicts a progression from articulation to action to outcomes. We know, however, that grand challenge trajectories are nonlinear processes with multiple unforeseeable feedback loops. Future work should explicitly ‘theorize the arrows’ within this framework to inform the processes and relationships that drive and connect articulation, actions and outcomes and give rise to specific trajectories that either mitigate or amplify grand challenges.

The pandemic has also illustrated how grand challenges can ripple rapidly into other domains. What began as a global health crisis has triggered organizational and economic changes that have affected work as we know it – throwing up a host of questions about employee wellbeing, productivity and engagement, team functioning and culture, organizational strategy and innovation, and industry survival – topics that not only demand nuanced study to understand the mechanisms of tenacity and change, but that are also firmly in the *content* domain of organizational and management studies. These topics, in turn, raise further questions around inequality, gender, health, corporate responsibility, and so on.

This final lesson from the pandemic shows that, while societal grand challenges might have once seemed distant from our ‘lane’ as organizational scholars, they will increasingly unleash consequences that impinge directly on organizations and work. Let us roll up our sleeves and use our expertise with complexity and multilevel explanations to debate, inform and enable progress on these extraordinarily complex problems. After all, the original mathematical formulation of grand challenges was also an invitation to do work that mattered – and what can have more impact in a world turned upside down?

## Note

[1] Others use the term ‘wicked problems’ because it captures very similar characteristics (Reneicke and Ansari, 2016).
